# Zinc Oxide Nanoparticle Caused Plasma Metabolomic Perturbations Correlate with Hepatic Steatosis

**DOI:** 10.3389/fphar.2018.00057

**Published:** 2018-01-30

**Authors:** Weidong Zhang, Yong Zhao, Fuli Li, Lan Li, Yanni Feng, Lingjiang Min, Dongxue Ma, Shuai Yu, Jing Liu, Hongfu Zhang, Tianhong Shi, Fuwei Li, Wei Shen

**Affiliations:** ^1^College of Life Sciences, Qingdao Agricultural University, Qingdao, China; ^2^State Key Laboratory of Animal Nutrition, Institute of Animal Sciences, Chinese Academy of Agricultural Sciences, Beijing, China; ^3^Qingdao Institute of Bioenergy and Bioprocess Technology, Chinese Academy of Sciences, Qingdao, China; ^4^Core Laboratories of Qingdao Agricultural University, Qingdao, China; ^5^Institute of Poultry Science, Shandong Academy of Agricultural Sciences, Jinan, China

**Keywords:** zinc oxide nanoparticles, plasma, metabolomics, perturbation, hepatic steatosis

## Abstract

Zinc oxide nanoparticles (ZnO NPs), known for their chemical stability and strong adsorption, are used in everyday items such as cosmetics, sunscreens, and prophylactic drugs. However, they have also been found to adversely affect organisms; previously we found that ZnO NPs disrupt pubertal ovarian development, inhibit embryonic development by upsetting γ-H2AX and NF-κB pathways, and even disturb skin stem cells. Non-targeted metabolomic analysis of biological organisms has been suggested as an unbiased tool for the investigation of perturbations in response to NPs and their underlying mechanisms. Although metabolomics has been used in nanotoxicological studies, very few reports have used it to investigate the effects of ZnO NPs exposure. In the current investigation, through a metabolomics-based approach, we discovered that ZnO NPs caused changes in plasma metabolites involved in anti-oxidative mechanisms, energy metabolism, and lipid metabolism in hen livers. These results are in line with earlier findings that ZnO NPs perturb the tricarboxylic acid cycle and in turn result in the use of alternative energy sources. We also found that ZnO NPs disturbed lipid metabolism in the liver and consequently impacted blood lipid balance. Changes in plasma metabolomes were correlated with hepatic steatosis.

## Introduction

Metabolomics is considered a useful tool for environmental risk assessment (Ryan and Robards, 2006; Taylor et al., 2010). Furthermore, non-targeted metabolomic analysis of biological organisms has been suggested as an unbiased tool for the investigation of perturbations in response to environmental toxicants and underlying mechanisms (Bundy et al., 2009; Garcia-Contreras et al., 2015; Gioria et al., 2016). Although metabolomics has been used in nanotoxicological studies (Parveen et al., 2012; Lv et al., 2015; Boyles et al., 2016; Carrola et al., 2016), its application has been limited to metabolites from exposure to TiO_2_ and SiO_2_ nanoparticles (NPs), and to a lesser extent, to silver, zinc, and copper NPs, and carbon nanotube (CNT) materials (Lv et al., 2015).

Zinc oxide (ZnO) NPs are commonly used due to their chemical stability and strong adsorption characteristics; they are the third most highly produced NPs in the world and are used in cosmetics, sunscreens, and as semiconductors and elements in medical or environmental science (Wang, 2008; Zhao et al., 2009). Moreover, ZnO NPs have been used in prophylactic drugs against bacterial diseases due to their antibacterial activity (Yan et al., 2012). Reports have shown, however, that they can adversely affect organisms such as mice (Bargheer et al., 2015; Yang et al., 2015) and rats (Hong et al., 2014a,b; Choi et al., 2015), and also human cells (Kim et al., 2013; Tuomela et al., 2013). In our previous reports, we have described how ZnO NPs disrupted pubertal hen ovarian development (Liu et al., 2016), inhibited chick embryonic development by upsetting the γ-H2AX and NF-κB pathways (Liu et al., 2017), and even disturbed skin stem cells (Ge et al., 2017). Although metabolomics has been used in nanotoxicological studies, only a few researchers have investigated the effects of ZnO particle exposure. Yan et al. (2012) used this tool to reveal nephrotoxicity in rats after a 14-consecutive day oral administration of 50 nm ZnO NPs. Moreover, Lee et al. (2016) identified respiratory toxicology after ZnO NP inhalation. Even though these two studies investigated metabolome change after ZnO NP administration, the treatment time was relatively short (1–14 days) and they only studied metabolomic alterations in the kidneys, lungs, and bronchoalveolar lavage fluid (BALF). Systemic metabolome perturbation in animal blood has not yet been studied.

In the current investigation, we aimed to explore metabolomic alterations following short-term (4 weeks) and long-term (24 weeks) exposure to different concentrations of orally administrated ZnO NPs. ZnSO_4_ was used to further investigate the impact of ZnO NPs on metabolomes, to determine their origin from either Zn^2+^ or intact NPs. The low molecular weight metabolites (LMWM) model and the lipoprotein lipid and albumin (LIPO) model were used to fully explore both large and small molecule metabolites disturbed by ZnO NPs. The secondary aim was to further investigate the possible molecular events underlying ZnO NP-induced systemic effects.

## Materials and Methods

### Characterization of ZnO NPs

Zinc oxide nanoparticles were synthesized by Beijing DK Nano Technology Co. Ltd. (Beijing, China) as reported previously (Liu et al., 2016, 2017; Zhao et al., 2016a,b; Ge et al., 2017). The characteristics of ZnO NPs (morphology, size, agglomeration, etc.) were determined by transmission electron microscopy (TEM; JEM-2100F, JEOL Inc., Japan) and dynamic light scattering (DLS) particle size analyzer (Nano-Zetasizer-HT, Malvern Instruments, Malvern, United Kingdom).

### Animal Study Design (Diets and Treatments) and Sample Collection

This investigation was performed in strict accordance with the recommendations in the Guide for the Care and Use of Laboratory Animals of the National Institutes of Health. The protocol (protocol number: QAU20161142) was approved by the Committee on the Ethics of Animal Experiments of Qingdao Agricultural University Institutional Animal Care and Use Committee (IACUC) (Zhao et al., 2016a,b; Liu et al., 2017). All hens (Jinghong-1 strain) were housed in a ventilated and conventional caged commercial poultry house with a lighting program of 16:8 light/dark and *ad lib* food and water. The formulation of the basal diet (corn–soybean base) has been previously reported (**Supplementary Table S1**) (Zhao et al., 2016a,b). There were seven treatments: (Taylor et al., 2010) Control treatment (no Zn added); (Ryan and Robards, 2006) ZnSO_4_-25 mg/kg; (Gioria et al., 2016) ZnSO_4_-50 mg/kg; (Garcia-Contreras et al., 2015) ZnSO_4_-100 mg/kg; (Bundy et al., 2009) ZnO-NP-25 mg/kg; (Boyles et al., 2016) ZnO-NP-50 mg/kg; and (Carrola et al., 2016) ZnO-NP-100 mg/kg. The concentrations of ZnO NPs or ZnSO_4_ used in our studies were based on the diet. If the concentration of 100 mg/kg of diet was calculated based on animal body weight (BW), it was calculated to be around 10 mg/kg BW. Therefore, the current concentrations were lower than those used in other studies (100–1000 mg/kg BW) (Yan et al., 2012; Hong et al., 2014a,b). A total of 420 hens were randomly assigned into the seven treatments, with three replicates per treatment and 20 hens per replicate. Experimental feeding started at 6 weeks (wks) of age. After 4 or 24 wks of exposure, 12 hens from each treatment were humanely slaughtered and blood (plasma) and tissue/organ samples were collected and stored at -80°C.

### Detection of ZnO NPs in Liver Using Transmission Electron Microscopy (TEM) and Energy Disperse Spectroscopy (EDS)

Sample preparation procedures for detecting NPs have been reported in our recent publication (Zhao et al., 2016a,b; Ge et al., 2017; Liu et al., 2017). Briefly, tissue samples were collected and fixed for 2 h in 2% glutaraldehyde made in sodium phosphate buffer (pH 7.2). Specimens were then washed extensively to remove the excess fixative and subsequently post-fixed in 1% OsO_4_ for 1 h in the dark. Specimens were then dehydrated in an increasingly graded series of ethanol and infiltrated with increased concentrations of Spur’s embedding medium in propylene epoxide. Subsequently, the specimens were polymerized in embedding medium for 12 h at 37°C, 12 h at 45°C, and 48 h at 60°C. Fifty nanometer sections were cut on a Leica Ultracut E microtome equipped with a diamond knife (Diatome, Hatfield, PA, United States), and collected on form var-coated, carbon-stabilized Mo grids. The section containing grids were stained with uranyl acetate, air dried overnight, and imaged on a JEM-2010F TEM (JEOL Ltd., Japan). The presence of ZnO NPs in the tissues was confirmed by using X-Max^N^ 80 TLE EDS (Oxford Instruments, United Kingdom).

### NMR Spectroscopy

Nuclear magnetic resonance (NMR) analyses were performed as previously described with slight modifications (Soininen et al., 2009; Lee et al., 2016). Before the NMR spectroscopy, 200 μl plasma was mixed with 80 μl D_2_O solution containing sodium phosphate buffer (0.1 M, pH 7.4) and sodium 3-trimethylsilyl-2,2, 3,3-d4-propionate (TSP) as an internal standard (δ = 0 ppm). The ^1^H NMR spectra was acquired using a 600.13 MHz Bruker AV600 spectrometer (Bruker, Rheinstetten, Germany) with a 5-mm CryoProbe at 300 K. NOESY and a zg pulse sequence of ^1^H NMR spectra and zggpr pulse sequence of J-resolved (JRES) NMR spectra were used to acquire the NMR information. In plasma NMR analyses, molecular identification and quantification are hampered by the complexity of plasma samples that contain a wide variety of molecules. To resolve this issue, we adopted an approach based on two molecular models, the LMWM model and the LIPO model, as previously described (Soininen et al., 2009). The LIPO model provides information on lipoprotein lipids and subclasses which are acquired through water-suppressed ^1^H NMR spectrum of serum. Alternately, the LMWM model is acquired by suppression of most of the broad macromolecules and lipoprotein lipid signals; this improves the sensitivity of low-molecular-weight metabolites (Mäkinen et al., 2008; Soininen et al., 2009; Yan et al., 2012; Wan et al., 2016). The LIPO window showing broad overlapping ^1^H NMR resonances coming mainly from lipid molecules in various lipoprotein particles were recorded with 80 k data points after four dummy scans using eight transients acquired with an automatically calibrated 90° pulse and applying a Bruker NOESY presat pulse sequence with mixing time of 10 ms and irradiation field of 25 Hz to suppress the water peak. The acquisition time was 2.7 s and the relaxation delay 3.0 s. The 90° pulse was calibrated automatically for each sample. A constant receiver gain setting was applied for all the samples. The LMWM data were acquired using a *T*_2_-relaxation-filtered pulse sequence which suppressed most of the broad macromolecule and lipoprotein lipid signals and enhanced the detection smaller molecules. The LMWM data were recorded with 64 k data points using 24 (or 16) transients acquired after four steady-state scans with a Bruker 1D CPMG pulse sequence with water peak suppression and a 78 ms *T*_2_-filter with a fixed echo delay of 403 ms to minimize diffusion and J-modulation effects. The acquisition time was 3.3 s and the relaxation delay 3.0 s. Both LIPO and LMWM data were processed and phase corrected in an automated fashion. Prior to Fourier transformations to spectra, the measured free induction decays for both LIPO and LMWM windows were zero-filled to 128 k data points and then multiplied with an exponential window function with a 1.0 Hz line broadening.

### NMR Spectral Processing and Analysis

The ^1^H NMR spectra were processed by MestRe-C2.3 software (Yan et al., 2012). The spectra were binned with a unit of 0.005 ppm between 0.2 and 10.0 ppm, and then integrated spectral intensity for each bin. The regions of internal standard and water resonance were excluded before being normalized by the total spectral area. The binned data were adjusted by generalized log transformation and mean-centered before multivariate analysis.

### Multivariate Analyses

The processed NMR datasets were examined by using principal component analysis (PCA) and partial least squares discriminant analysis (PLS-DA) by SIMCA-P10.0 software package (Version 10, Umetrics AB, and Umea, Sweden). PCA was used to reduce the complexity of the metabolomics data matrix without additional information and provides the visual performance of the original cluster for each group. PLS-DA connected the classified information and NMR dataset to determine the variance between the different treatment groups. Two-dimensional score plots were used to visualize the separation of the samples and the corresponding loading plots were applied to identify the spectral variable contribution to the position of the spectra that were altered by different treatments (Yan et al., 2012).

### qRT-PCR

Total RNA was isolated as described previously (Zhao et al., 2016a). RNA concentration was determined by Nanodrop 3300 (ThermoScientific, Wilmington, DE, United States). Two micrograms of total RNA was used to make the first-strand complementary DNA (cDNA; in 20 μl) using RT2 First Strand Kit (Cat. No: AT311-03, Transgen Biotech, China) following the manufacturer’s instructions. The generated first-strand cDNAs (20 μl) was diluted to 150 μl with double-deionized water (ddH_2_O). Then, 1 μl was used for one PCR reaction (in a 96-well plate). Each PCR reaction (12 μl) contained 6 μl of qPCR Master Mix (Roche, German), 1 μl of diluted first-stand cDNA, 0.6 μl primers (10 mM), and 4.4 μl of ddH_2_O. The primers for qPCR analysis were synthesized by Invitrogen and present in **Supplementary Table S2**. The qPCR was conducted by the Roche LightCycler^®^ 480 (Roche, German) with the following program – step 1: 95°C, 10 min; step 2: 40 cycles of 95°C, 15 s; 60°C, 1 min; step 3: dissociation curve, step 4: cool down. Three or more independent experiment samples were analyzed (Zhao et al., 2016a,b).

### Western Blotting

Liver samples were lysed in RIPA buffer containing a protease inhibitor cocktail from Sangon Biotech, Ltd. (Shanghai, China). Protein concentration was determined using a BCA kit (Beyotime Institute of Biotechnology, Shanghai, China) (Liu et al., 2017). The information for the primary antibodies (Abs) is present in **Supplementary Table S2**. GAPDH and Actin were used as loading controls. Secondary donkey anti-goat Ab (Cat no. A0181) was purchased from Beyotime Institute of Biotechnology, and goat anti-rabbit (Cat no.: A24531) Abs were bought from Novex^®^ by Life Technologies (United States). Fifty micrograms of total protein per sample was loaded onto 10% SDS polyacrylamide electrophoresis gels. The gels were transferred to a polyvinylidene fluoride (PVDF) membrane at 300 mA for 2.5 h at 4°C. Subsequently, the membranes were blocked with 5% bovine serum albumin (BSA) for 1 h at room temperature (RT), followed by three washes with 0.1% Tween-20 in TBS (TBST). The membranes were incubated with primary Abs (**Supplementary Table S3**) diluted at 1:500 in TBST with 1% BSA overnight at 4°C. After three washes with TBST, the blots were incubated with the HRP-labeled secondary goat anti-rabbit or donkey anti-goat Ab, respectively, for 1 h at RT. After three washes, the blots were imaged. The images were quantified by Image J.

### Statistical Analyses

The qRT-PCR data were statistically analyzed based on ΔΔC_t_ using proprietary software from SABiosciences online support^1^. Other data were statistically analyzed with SPSS statistics software (IBM Co., New York, NY, United States) using ANOVA. Comparisons between groups were tested by one-way ANOVA analysis and LSD tests. All groups were compared with each other for every parameter (mean ± SD). Differences were considered significant at *p* < 0.05.

## Results

### ZnO Nanoparticle Characterization

The ultra-structure of ZnO NPs used in this investigation has been published in our previous articles (**Supplementary Figure S1**) (Zhao et al., 2016a,b; Ge et al., 2017; Liu et al., 2017). The particles (∼30 nm) were almost spherical with a milk-white color, a surface area of 50 m^2^/g, and a density of 5.606 g/cm^3^. Intact NPs were identified in the liver (**Supplementary Figure S1**) by TEM and confirmed by energy dispersive spectroscopy (EDS) with Zn (**Supplementary Figure S1**). Three standard Zn peaks were noted.

### Effects of ZnO NPs on Plasma Metabolome

**FIGURE 1:**
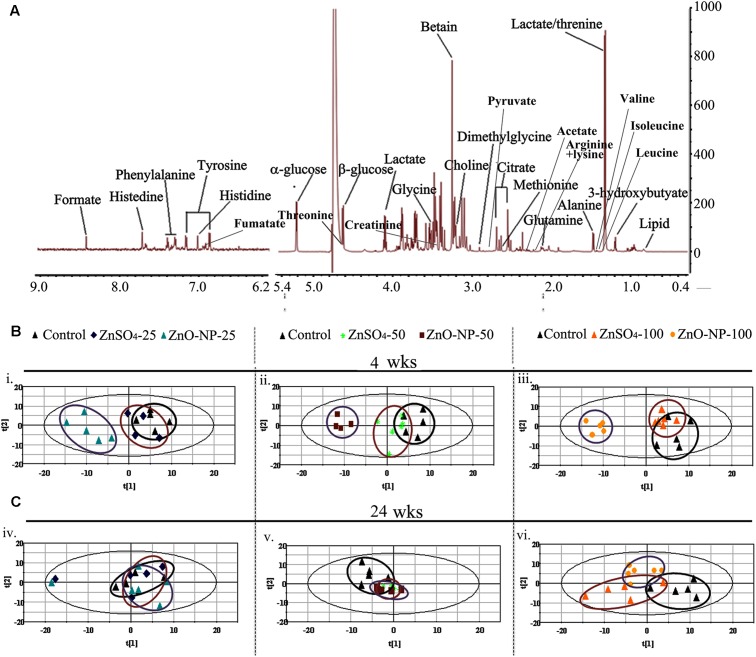
^1^H nuclear magnetic resonance (NMR) analysis of small molecule metabolites. **(A)** The NMR spectral characteristics and the metabolic contents of the low molecular weight metabolites (LMWM) model analysis. **(B)** The score plot of the principal component analysis (PCA) model from the analysis of ^1^H NMR spectra of plasma from hens exposed to ZnO nanoparticles (NPs), ZnSO_4_ (*n* ≥ 6 for each dose group) after a 4-week exposure. **(C)** The score plot of the PCA model from the analysis of ^1^H NMR spectra of plasma from hens exposed to ZnO NPs, ZnSO_4_ (*n* ≥ 6 for each dose group) after a 24-wk exposure.

**FIGURE 2:**
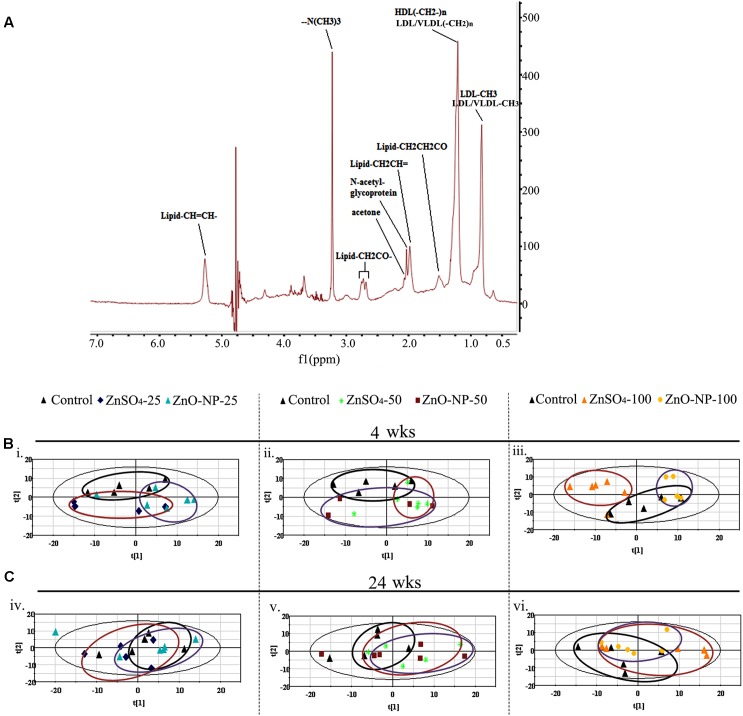
^1^H NMR analysis of large molecule metabolites. **(A)** The NMR spectral characteristics and the metabolic contents of the lipoprotein lipid and albumin (LIPO) model analysis. **(B)** Score plot of the PCA model from the analysis of ^1^H NMR spectra of plasma from animals exposed to ZnO NPs, ZnSO_4_ (*n* ≥ 6 for each dose group) after a 4-wk exposure. **(C)** The score plot of the PCA model from the analysis of ^1^H NMR spectra of plasma from animals exposed to ZnO NPs, ZnSO_4_ (*n* ≥ 6 for each dose group) after a 24-wk exposure.

The LMWM ^1^H NMR spectra, displaying metabolic fingerprints of small molecules from plasma metabolites of ZnO NP or ZnSO_4_-treated animals, are presented in **Figure 1A**; while the LIPO ^1^H NMR spectra, displaying metabolic fingerprints of large molecules from plasma metabolites of ZnO NPs or ZnSO_4_-treated animals, are presented in **Figure 2A**. Peaks were assigned to specific metabolites based on chemical shift and peak multiplicity according to previous literature (Mäkinen et al., 2008; Soininen et al., 2009; Yan et al., 2012; Wan et al., 2016). ZnSO_4_ was used in this investigation to compare the effects of either Zn^2+^ or intact particles because ZnSO_4_ produces a sole Zn^2+^ effect (**Supplementary Figure S2**).

Partial least squares discriminant analysis was used to uncover latent biochemical information from the ^1^H NMR spectra. In the LMWM model for small molecules, the score plots showed a clear separation between ZnO NPs and ZnSO_4_ at 25, 50, and 100 mg/kg after 4 wks of exposure (**Figures 1B,C**). Furthermore, there was a clear separation between ZnO NP treatment and the control group, but not between the ZnSO_4_ treatment and the control. After 24 wks of exposure, there was no separation between ZnO NPs, ZnSO_4_, or the control (**Figures 1B,C**). In the LIPO model for large molecules, the score plots showed an unclear separation between ZnO NPs, ZnSO_4_, and the control after 4 or 24 wks of exposure (**Figures 2B,C**).

**FIGURE 3:**
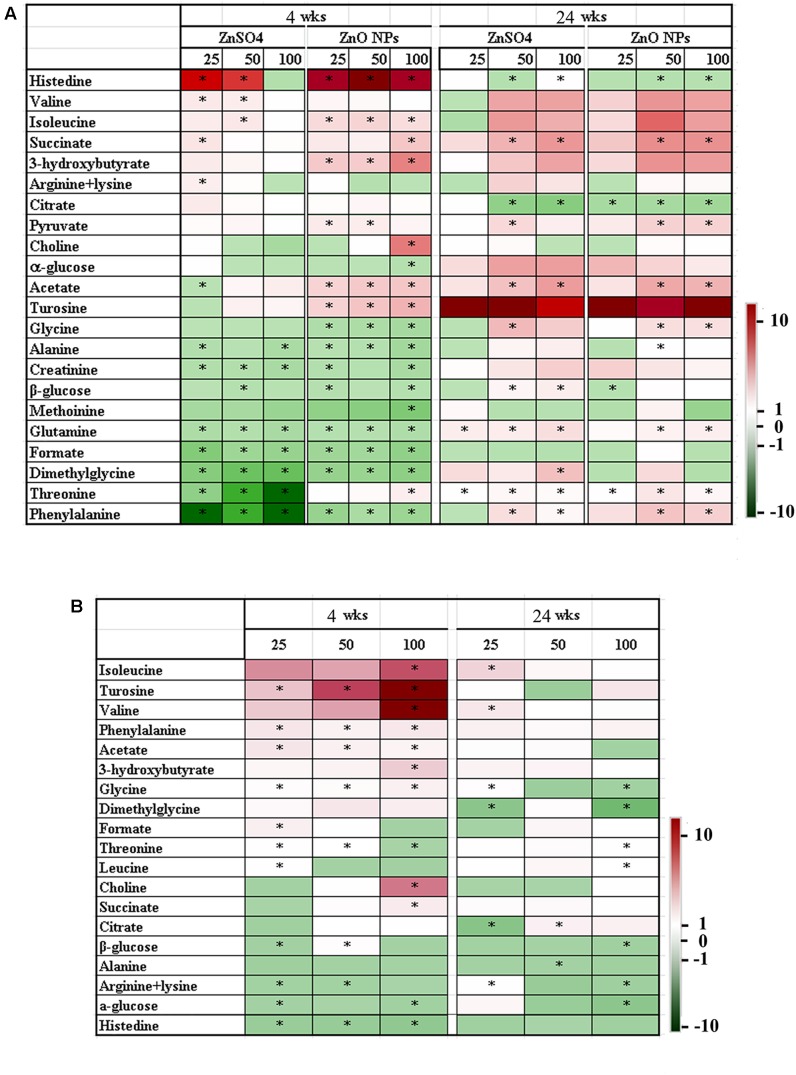
Heatmap of the main small molecule metabolite variations in plasma samples. **(A)** Data from different concentrations of ZnO NPs or ZnSO_4_ exposure compared to the control. **(B)** Data from different concentrations of ZnO NPs compared to ZnSO_4_ (^∗^*p* < 0.05; *n* ≥ 6).

A number of perturbations in endogenous metabolites were observed in the ^1^H NMR spectra of plasma samples in both the LMWM and LIPO models. **Figure 3** presents prominent small molecule changes in the LMWM model analysis between ZnO NP exposure and the control, or between ZnSO_4_ exposure and the control. Compared to the control, 23 metabolites were altered (**Figure 3A**); of these, 12 amino acids: histidine, valine, isoleucine, tyrosine, glycine, alanine, arginine (lysine), methionine, phenylalanine, threonine, and glutamine were altered (**Figure 3A**). The remaining 11 metabolites included fumarate, citrate, succinate, 3-hydroxybutyrate, acetate, pyruvate, formate, dimethylglycine, α-glucose, and β-glucose (**Figure 3A**). After 4 wks of exposure, when compared to the control, ZnO NPs produced more profound metabolite changes than ZnSO_4_ in a dose-dependent manner. Around half of the changed metabolites were reduced by ZnO NPs or ZnSO_4_ treatments. After 24 wks of treatment, less significant changes were found for both ZnO NPs and ZnSO_4_ exposure (**Figure 3A**). However, most of the changed metabolites were elevated compared to the control by ZnO NPs or ZnSO_4_.

We also aimed to determine the effects of ZnO NPs on metabolites coming from Zn^2+^ or intact particles. Therefore, changes in metabolites were compared between ZnO NPs and ZnSO_4_ at different concentrations and different time points. Nineteen metabolites were differentially altered by ZnO NPs compared to ZnSO_4_ (**Figure 3B**). After 4 wks of exposure, about half of them showed a decrease and the other half were increased. The three most changed metabolites were isoleucine, tyrosine, and valine, which were dramatically elevated by ZnO NPs. After 24 wks of exposure, most metabolites were reduced by ZnO NPs (**Figure 3B**); the most changed metabolites were glycine, dimethylglycine, citrate, and glucose (all decreased). The data here suggested that ZnO NPs were different from ZnSO_4_ in that the intact NPs might play an important role in altering the levels of small molecule metabolites.

**FIGURE 4:**
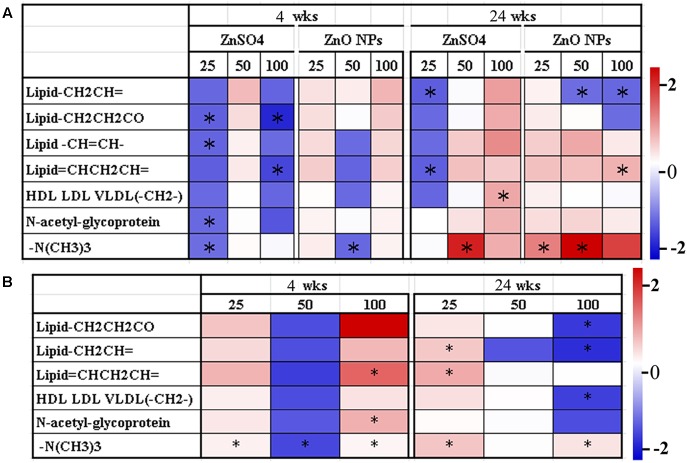
Heatmap of the main large molecule metabolite variations in plasma samples. **(A)** Data from different concentrations of ZnO NPs or ZnSO_4_ exposure compared to the control. **(B)** Data from different concentrations of ZnO NPs compared to ZnSO_4_ (^∗^*p* < 0.05; *n* ≥ 6).

**Figure 4A** shows the prominent large molecule changes in the LIPO model analysis between ZnO NP exposure and the control or between ZnSO_4_ exposure and the control. Compared to the control, six kinds of lipid molecules (Lipid-CH_2_CH=, Lipid-CH_2_CH_2_CO, Lipid-CH=CH-, Lipid=CHCH_2_CH=, HDL LDL VLDL(-CH_2_-), and -N(CH_3_)_3_] and one kind of protein (*N*-acetyl-glycoprotein) were changed by ZnO NPs or ZnSO_4_ exposure (**Figure 4A**). After 4 wks of exposure, ZnSO_4_ produced more profound effects on these large metabolites than ZnO NPs. However, after 24 wks of exposure, ZnO NPs produced more profound effects on these large metabolites than ZnSO_4_. Compared to ZnSO_4_, ZnO NPs differentially altered the levels of these large metabolites at the two experimental time points (**Figure 4B**). After 4 wks of exposure, ZnO NPs increased Lipid–CH=CH–, –N(CH_3_)_3_, and *N*-acetyl-glycoprotein. After 24 wks of exposure, the 25 mg/kg ZnO NPs treatment increased Lipid–CH=CH–, Lipid=CHCH_2_CH=, Lipid–CH=CH–, and –N(CH_3_)_3_ compared to the 25 mg/kg ZnSO_4_ exposure; however, Lipid–CH_2_CH_2_CO, Lipid–CH_2_CH=, and HDL LDL VLDL(–CH_2_–) were lower in the 100 mg/kg ZnO NP exposure than that in the 100 mg/kg ZnSO_4_ exposure (**Figure 4B**). The data here further indicated that the effect of ZnO NPs was different from that of ZnSO_4_ in that the intact NPs might play an important role in changing the levels of large molecule metabolites.

### ZnO NPs Altered Liver Metabolism Enzymes

**FIGURE 5:**
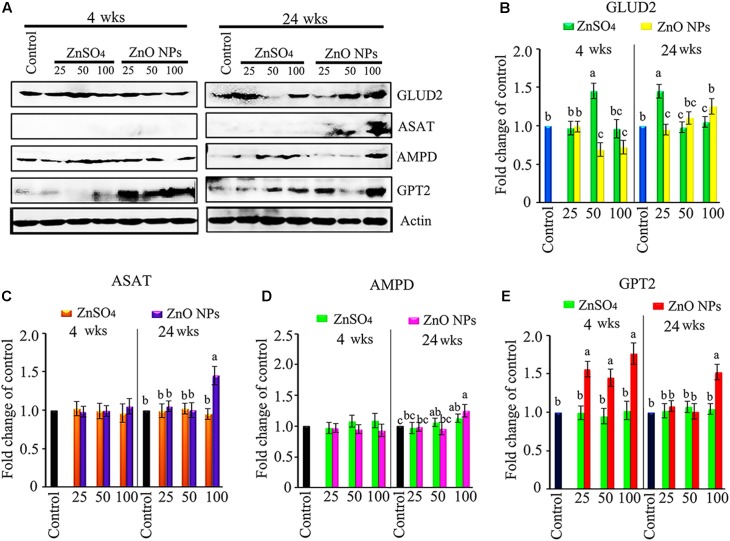
Stimulation of amino acid metabolism enzymes by ZnO NPs. **(A)** Elevation in GLUD2, ASAT, AMPD, and GPT2 by ZnO NPs using western blotting (WB) analysis. **(B)** WB quantitative data for GLUD2. **(C)** WB quantitative data for ASAT. **(D)** WB quantitative data for AMPD. **(E)** WB quantitative data for GPT2. Data present as average ± SEM. a, b, c indicate a significant difference among different treatments (*p* < 0.05; *n* ≥ 6).

In order to explore the underlying mechanisms of metabolomic changes caused by ZnO NPs, metabolic enzymes in the liver were investigated. Glutamate dehydrogenase (GLUD2) and aspartate aminotransferase (ASAT) are two important enzymes associated with amino acid metabolism. Compared to the control, 4 wks of 50 mg/kg ZnSO_4_ exposure increased GLUD2; however, 50 and 100 mg/kg ZnO NP exposure decreased GLUD2 protein level (**Figure 5**). After 24 wks, both 25 mg/kg ZnSO_4_ and 100 mg/kg ZnO NPs increased GLUD2. ASAT was increased by 100 mg/kg ZnO NP exposure for 24 wks. AMP deaminase (AMPD) is a vital enzyme for AMP metabolism and plays a critical role in energy metabolism. GPT2 catalyzes a reversible transamination reaction to yield glutamate and pyruvate, and participates in amino acid metabolism and gluconeogenesis. AMPD was increased by the 100 mg/kg ZnO NP treatment after 24 wks of exposure. After 4 wks, GPT2 was elevated by the 25, 50, and 100 mg/kg ZnO NPs exposure. After 24 wks, GPT2 was stimulated by the 100 mg/kg ZnO NPs exposure (**Figure 5**). Several other liver metabolism enzymes such as UGT, β-oxidation, CYP2A, CYP2B, and ACACA were analyzed, but they remained unaffected by either ZnSO_4_ or ZnO NPs. The data here suggest that ZnO NPs differentially affected liver metabolism enzymes as compared to ZnSO_4_.

### Liver Histopathology

**FIGURE 6:**
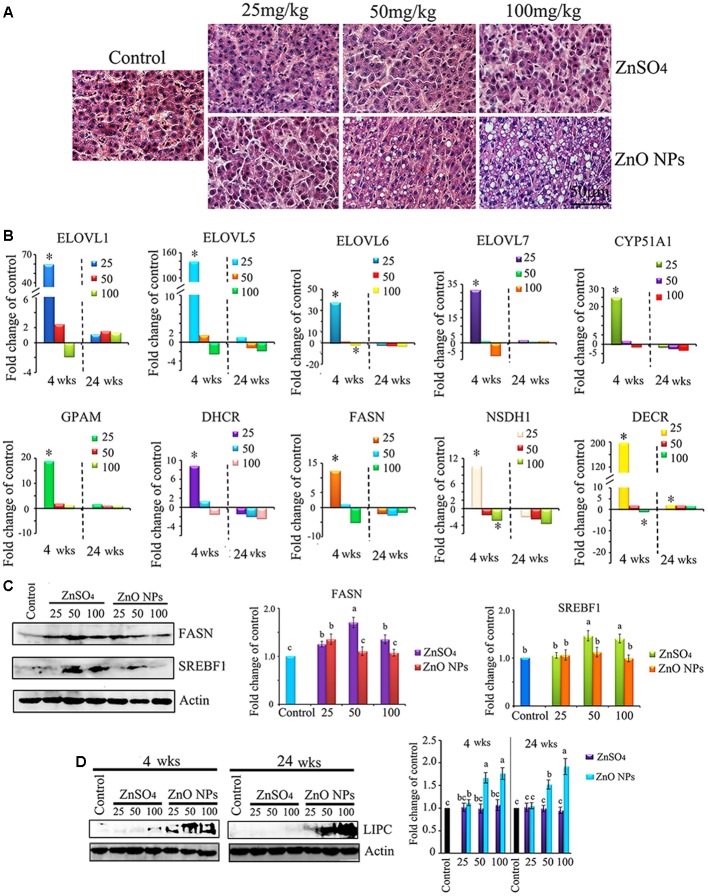
Effect of ZnO NPs on liver lipid metabolism enzymes. **(A)** H&E staining of liver sections showing the variable size of fat vacuoles in ZnO NP exposure groups after 24 wks of treatment. **(B)** Gene expression of lipid synthesis enzymes compared to the control. **(C)** Alteration in protein levels of the lipid synthesis enzyme FASN and SREBF1. **(D)** Elevation of hepatic lipase (LIPC) by ZnO NPs after 4 or 24 wks of exposure. Data present as average ± SEM. a, b, c indicate a significant difference among different treatments (*p* < 0.05; *n* ≥ 6).

After periods of 4 or 24 wks exposure, no treatments affected BW. After 24 wks, ZnO NPs dose-dependently caused liver steatosis. As shown in **Figure 5A**, liver histopathology in ZnSO_4_ treatments was similar to that in the control group, while the grade of macrovesicular liver steatosis in the 50 and 100 mg/kg ZnO NP exposure groups was increased with an elevation of relative liver weight in these groups (**Figure 6A**; Hanin et al., 2017).

### ZnO NPs Disrupted Liver Lipid Metabolism Enzymes’ Gene or Protein Expression

It was suggested that liver lipid metabolism enzymes might be disturbed by ZnO NPs since plasma lipid levels were differentially altered. Therefore, gene expression of fatty acid synthesis enzymes and lipid synthesis enzymes were investigated. It was found that, after 4 wks, the 25 mg/kg ZnO NP exposure stimulated the gene expression of ELOVL1, ELOVL5, ELOVL6, ELOVL7, CYP51A1, GPAM, DHCRT, FASN, NSDH1, and DECRL, which matched the metabolomics data (**Figure 6B**). However, after 24 wks, most of these genes were decreased by ZnO NP exposure compared to the control (**Figure 6B**). The protein levels of important lipid synthesis enzymes were also investigated and it was found that FASN and SREBF1 were altered. After 24 wks, FASN was stimulated by the 25, 50, and 100 mg/kg ZnSO_4_ treatments; however, it was only increased by the 25 mg/kg ZnO NPs. After 24 wks, SREBF1 was elevated by the 50 and 100 mg/kg ZnSO_4_ exposure but not by ZnO NPs (**Figure 6C**). Hepatic lipase (LIPC) was also explored. LIPC was elevated by the 50 and 100 mg/kg ZnO NP treatments after 4 and 24 wks of exposure. The data here suggested lipid degradation might be stimulated by ZnO NPs, and lipid synthesis might be decreased by ZnO NPs (**Figure 6D**). The data in this section indicated that lipid metabolites in plasma might reflect lipid metabolism in the livers under ZnO NP treatment.

### ZnO NPs Caused Liver Damage and Apoptosis

**FIGURE 7:**
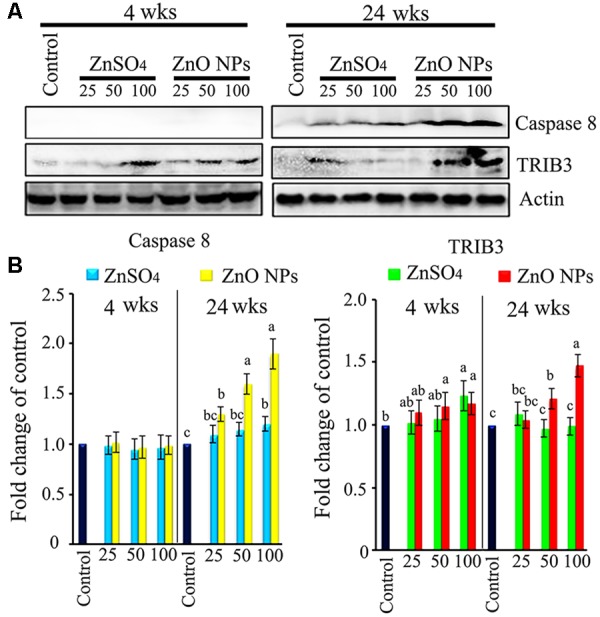
Liver cell damage and apoptosis caused by ZnO NPs. **(A)** WB image for cell damage marker TRIB3 and cell apoptosis marker caspase 8 in liver tissue. **(B)** Quantitative data for WB. Data present as average ± SEM. a, b, c indicate a significant difference among different treatments (*p* < 0.05; *n* ≥ 6).

Liver damage and apoptosis were investigated because liver function was altered by ZnO NP treatment. We found that the apoptosis marker caspase 8 was elevated by the 25, 50, and 100 mg/kg ZnO NP treatments after 24 wks of exposure in a dose-dependent manner. Mitochondrial damage marker TRIB3 was increased by the 50 and 100 mg/kg ZnO NP treatments after 4 and 24 wks exposure (**Figure 7**). Apoptosis markers caspase 3, Bcl-2, Bcl-xl, Bax, p53, and damage marker DDIT3 were also investigated; however, they remained unaltered by ZnO NPs. The data here suggest that the ZnO NPs might have caused the death of liver cells.

## Discussion

Yan et al. (2012) reported the metabolic effects of oral administration of ZnO NPs on rat kidneys, while Lee et al. (2016) demonstrated that the inhalation of ZnO NPs and fine-sized particles altered the metabolome in rat lungs and BALF using NMR-based metabolomics. In Yan’s study, 50 nm ZnO NPs were administrated for 14 days and they disrupted energy metabolism and impaired cell membranes in rat kidneys. In Lee’s study, fine-sized or ZnO NPs were administered by inhalation and acutely induced rat lung metabolic alterations. In these two studies, the treatments were short-term and metabolic changes in kidneys and lungs were investigated; however, the impact of these particles on the systemic metabolome of blood samples is, as yet, unknown. Furthermore, the effect of relative long-term exposure on metabolomes and the underlying mechanisms are not understood. In our previous studies, we found that pubertal hen’s ovarian development was perturbed (Liu et al., 2016), chicken embryonic development was inhibited (Liu et al., 2017), and even skin stem cells were disturbed by 30 nm ZnO NPs (Ge et al., 2017). The adverse effects of ZnO may be due to both intact particles and Zn^2+^. In the current investigation, 30 nm ZnO NPs were orally administrated for 4 or 24 wks and differences in plasma metabolites were explored. At the same time ZnSO_4_ was used to offer a comparison of the effects of ZnO NPs on the metabolome. It was found that the alteration in metabolites caused by ZnO NPs was different from that caused by ZnSO_4_, even though there was some overlap. This further confirms that the toxic effects of ZnO NPs come from both intact particles and released Zn^2+^.

After short-term exposure (4 wks), there was a greater change in small molecule metabolites due to ZnO NPs or ZnSO_4_ exposure, as compared to the control group, than that after long-term exposure (24 wks; **Figure 3**). In the PCA plots, ZnO NP treatment was clearly separated from ZnSO_4_ or the control group after 4 wks of treatment; however, ZnO NPs, ZnSO_4_, and the control group were mixed together in the PCA plots after 24 wks of exposure (**Figures 1B**, **2B**). These data matched those of previous studies in that acute exposure caused extensive alteration to metabolomes, which may be because ZnO NPs produce stress on organisms. However, the organisms may have feedback mechanisms to compensate for such stresses.

When compared to the control, most of the altered small molecule metabolites were decreased by ZnO NPs or ZnSO_4_ after 4 wks of exposure, while most of the altered small molecule metabolites were increased by ZnO NPs or ZnSO_4_ after 24 wks of exposure (**Figure 3**). When compared to ZnSO_4_, a greater number of small molecule metabolites were changed after 4 wks of ZnO NP exposure than after 24 wks of exposure and most of the altered small molecule metabolites were increased after the 4 wk ZnO NP exposure and were decreased after the 24 wk ZnO NP exposure.

Glutathione is a vital biological antioxidant which is formed by three amino acids glutamic acid, cysteine, and glycine (Biswas and Rahman, 2009). Glycine was decreased by 4 wks of ZnO NP exposure, which suggested that its production might be a protective response to oxidative stress cause by ZnO NPs. This was consistent with earlier observations that ZnO NPs might cause oxidative stress (Xia et al., 2008; Ho et al., 2011; Pujalte et al., 2011; Lee et al., 2016). However, the organism may use other feedback mechanisms in response to oxidative stress. Taurine, with its protective effects against oxidative stress, was elevated by 4 wks of ZnO NP exposure, compared to the control or ZnSO_4_ (Banks et al., 1991; Gurer et al., 2001; Schuller-Levis and Park, 2003; Schaffer et al., 2009). The data indicated that ZnO NPs caused oxidative stress systemically and on the other hand the organism used anti-oxidative pathways to defend against stress (Lee et al., 2016).

Many energy-related metabolites were disturbed by ZnO NPs. In the current investigation, after 4 wks of treatment, blood glucose (a-glucose and b-glucose) and alanine were reduced which indicated that aerobic metabolism and the tricarboxylic acid (TCA) cycle were perturbed in the liver. It is reported that Zn^2+^ released from ZnO NPs inhibits enzymes in the TCA cycle resulting in a reduction in citrate (Yan et al., 2012). We found similar results, that blood citrate was decreased after 24 wks of ZnO NP exposure compared to the control or ZnSO_4_. Isoleucine, valine, 3-hydroxybutyrate, and acetate are also related to energetic pathways in organisms. These four metabolites in blood were elevated by ZnO NPs compared to the control or ZnSO_4_ exposure in the current investigation; this agreed with the findings of Lee et al. (2016).

Branched chain amino acids (BCAA) are considered to be essential amino acids because they are not synthesized by animal bodies (Zhai et al., 2010), and any increase in BCAA may be due to increase in protein digestion. BCAA are also used as energy sources by organism (Fabisiak et al., 2011). Leucine, isoleucine, and valine were elevated by ZnO NPs compared to that in control or ZnSO4 after 4 or 24 wks of exposure in the current investigation. At the same time, the amino acid metabolism enzymes GLUD2 and ASAT in the liver were disrupted by ZnO NPs.

It has been reported by a few metal oxide toxicity studies that 3-hydroxybutyrate was increased in the serum or urine while glucose levels were reduced or the TCA cycle was disrupted (Lei et al., 2008; Bu et al., 2010; Yan et al., 2012). Moreover, 3-hydroxybutyrate, produced by the liver, is also considered as a marker of cell damage (Fabisiak et al., 2011). In the current investigation we found that plasma 3-hydroxybutyrate was elevated by ZnO NPs and at the same time apoptosis markers in the liver were elevated, as compared to the control or ZnSO_4_ exposure. After 24 wks of treatment, ZnO NPs caused hepatic steatosis. In addition, most blood lipids were decreased by ZnO NPs as compared to the control or ZnSO_4_ after 24 wks of exposure. Moreover, most lipid synthesis enzyme gene expression was decreased, even though the decrease was not significant. ZnSO_4_ increased the lipid synthesis enzymes FASN and SREBF1 protein levels; however, these proteins were not altered by ZnO NPs. Levels of hepatic LIPC were increased by ZnO NPs. These data suggested that ZnO caused the accumulation of lipids in the liver in which fewer lipids were released into the blood.

## Conclusion

In summary, through using a metabolomics-based approach, we discovered that ZnO NPs caused changes in the levels of metabolites involved in anti-oxidative mechanisms, energy metabolism, and lipid metabolism in hen livers. And the changes were more dramatic in a dose-dependent manner in the short exposure period (4 wks) than in the long exposure period (24 wks). These results agreed with earlier investigations that ZnO NPs perturbed the TCA cycle and, in turn, resulted in the use of alternative sources for energy production. Lee et al. (2016) found that ZnO NPs or fine-sized particles also disrupted lipid metabolism in lung tissue. Our study showed that ZnO NPs disturbed lipid metabolism in livers and consequently disturbed blood lipid levels; and plasma metabolome alterations were correlated with hepatic steatosis.

## Author Contributions

WS, HZ, and YZ provided key intellectual input in the conception and design of these studies and aided in the writing of this manuscript. WZ, LL, and YF performed the animal experiments. LM, DM, SY, and JL performed the molecular experiments. FlL, FwL, and TS provided expertise for data explanation and contributed to the writing of the manuscript. All authors read and approved the final manuscript.

## Conflict of Interest Statement

The authors declare that the research was conducted in the absence of any commercial or financial relationships that could be construed as a potential conflict of interest.

## Footnotes

1 www.SABiosciences.com

## Supplementary Material

**FIGURE S1 |** ZnO NPs in liver. **(A)** TEM photograph of ZnO NPs and their characteristics. **(B)** TEM photograph of ZnO NPs in liver tissue is indicated by the white arrow. **(C)** EDS picture of ZnO NPs in liver, where three Zn peaks are shown (Zhao et al., 2016b).

**FIGURE S2 |** Effects of ZnO NPs and ZnSO_4_ treatments on blood Zn content. *Z*-axis represents the content (mg/kg wet mass), and the *Y*-axis represents the treatment (concentration of Zn; mg/kg) (^∗^*p* < 0.05, compared to the control; Zhao et al., 2016b).

**TABLE S1 |** Ingredient composition of the basal diet for the layers.

**TABLE S2 |** Primer sequences for q-RT-PCR.

**TABLE S3 |** Primary antibody information.

## References

[B1] Banks M. A. Porter D. W. Martin W. G. Castranova V. (1991). Ozone-induced lipid peroxidation and membrane leakage in isolated rat alveolar macrophages: protective effects of taurine. *J. Nutr. Biochem.* 2 308–313. 10.1016/0955-2863(91)90072-D

[B2] Bargheer D. Giemsa A. Freund B. Heine M. Waurisch C. Stachowski G. M. (2015). The distribution and degradation of radiolabeled superparamagnetic iron oxide nanoparticles and quantum dots in mice. *Beilstein J. Nanotechnol.* 6 111–123. 10.3762/bjnano.6.11 25671156 PMC4311637

[B3] Biswas S. K. Rahman I. (2009). Environmental toxicity, redox signaling and lung inflammation: the role of glutathione. *Mol. Aspects Med.* 30 60–76. 10.1016/j.mam.2008.07.001 18760298 PMC2699458

[B4] Boyles M. S. Ranninger C. Reischl R. Rurik M. Tessadri R. Kohlbacher O. (2016). Copper oxide nanoparticle toxicity profiling using untargeted metabolomics. *Part Fibre Toxicol.* 13 49120. 10.1186/s12989-016-0160-6 27609141 PMC5017021

[B5] Bu Q. Yan G. Deng P. Peng F. Lin H. Xu Y. (2010). NMR-based metabonomic study of the sub-acute toxicity of titanium dioxide nanoparticles in rats after oral administration. *Nanotechnology* 21 125105. 10.1088/0957-4484/21/12/125105 20203358

[B6] Bundy J. G. Davey M. P. Viant M. R. (2009). Environmental metabolomics: a critical review and future perspectives. *Metabolomics* 5 3–21. 10.1002/etc.3218 26771350

[B7] Carrola J. Bastos V. Jarak I. Oliveira-Silva R. Malheiro E. Daniel-da-Silva A. L. (2016). Metabolomics of silver nanoparticles toxicity in HaCaT cells: structure-activity relationships and role of ionic silver and oxidative stress. *Nanotoxicology* 10 1105–1117. 10.1080/17435390.2016.1177744 27144425

[B8] Choi J. Kim H. Kim P. Jo E. Kim H. M. Lee M. Y. (2015). Toxicity of zinc oxide nanoparticles in rats treated by two different routes: single intravenous injection and single oral administration. *J. Toxicol. Environ. Health A* 78 226–243. 10.1080/15287394.2014.949949 25674826

[B9] Fabisiak J. P. Medvedovic M. Alexander D. C. Mcdunn J. E. Concel V. J. Bein K. (2011). Integrative metabolome and transcriptome profiling reveals discordant energetic stress between mouse strains with differential sensitivity to acrolein-induced acute lung injury. *Mol. Nutr. Food Res.* 55 1423–1434. 10.1002/mnfr.201100291 21823223 PMC3482455

[B10] Garcia-Contreras R. Sugimoto M. Umemura N. Kaneko M. Hatakeyama Y. Soga T. (2015). Alteration of metabolomic profiles by titanium dioxide nanoparticles in human gingivitis model. *Biomaterials* 57 33–40. 10.1016/j.biomaterials.2015.03.059 25913073

[B11] Ge W. Zhao Y. Lai F. Liu J. Sun Y. Wang J. (2017). Cutaneous applied nano-ZnO reduce the ability of hair follicle stem cells to differentiate. *Nanotoxicology* 6 1–10. 10.1080/17435390.2017.1310947 28326861

[B12] Gioria S. Lobo Vicente J. Barboro P. La Spina R. Tomasi G. Urbán P. (2016). A combined proteomics and metabolomics approach to assess the effects of gold nanoparticles in vitro. *Nanotoxicology* 10 736–748. 10.3109/17435390.2015.1121412 26647645 PMC4898143

[B13] Gurer H. Ozgunes H. Saygin E. Ercal N. (2001). Antioxidant effect of taurine against lead-induced oxidative stress. *Arch. Environ. Contam. Toxicol.* 41 397–402. 10.1007/s002440010265 11598776

[B14] Hanin G. Yayon N. Tzur Y. Haviv R. Bennett E. R. Udi S. (2017). miRNA-132 induces hepatic steatosis and hyperlipidaemia by synergistic multitarget suppression. *Gut* 10.1136/gutjnl-2016-312869 [Epub ahead of print]. 28381526 PMC5969364

[B15] Ho M. Wu K. Y. Chein H. M. Chen L. C. Cheng T. J. (2011). Pulmonary toxicity of inhaled nanoscale and fine zinc oxide particles: mass and surface area as an exposure metric. *Inhal. Toxicol.* 23 947–956. 10.3109/08958378.2011.629235 22122307

[B16] Hong J. S. Park M. K. Kim M. S. Lim J. H. Park G. J. Maeng E. H. (2014a). Prenatal development toxicity study of zinc oxide nanoparticles in rats. *Int. J. Nanomedicine* 9(Suppl. 2), 159–171. 10.2147/IJN.S57932 25565834 PMC4279776

[B17] Hong J. S. Park M. K. Kim M. S. Lim J. H. Park G. J. Maeng E. H. (2014b). Effect of zinc oxide nanoparticles on dams and embryo-fetal development in rats. *Int. J. Nanomedicine* 9(Suppl. 2), 145–157. 10.2147/IJN.S57931 25565833 PMC4279755

[B18] Kim A. R. Ahmed F. R. Jung G. Y. Cho S. W. Kim D. I. Um S. H. (2013). Hepatocyte cytotoxicity evaluation with zinc oxide nanoparticles. *J. Biomed. Nanotechnol.* 9 926–929. 10.1166/jbn.2013.1495 23802425

[B19] Lee S. H. Wang T. Y. Hong J. H. Cheng T. J. Lin C. Y. (2016). NMR-based metabolomics to determine acute inhalation effects of nano- and fine-sized ZnO particles in rat lung. *Nanotoxicology* 10 924–934. 10.3109/17435390.2016.1144825 27245357

[B20] Lei R. Wu C. Yang B. Ma H. Shi C. Wang Q. (2008). Integrated metabolomic analysis of the nano-sized copper particle-induced hepatotoxicity and nephrotoxicity in rats: a rapid in vivo screening method for nanotoxicity. *Toxicol. Appl. Pharmacol.* 232 292–301. 10.1016/j.taap.2008.06.026 18706438

[B21] Liu J. Zhao Y. Ge W. Zhang P. Liu X. Zhang W. (2017). Oocyte exposure to ZnO nanoparticles inhibits early embryonic development through the γ-H2AX and NF-κB signaling pathways. *Oncotarget* 8 42673–42692. 28487501 10.18632/oncotarget.17349 PMC5522097

[B22] Liu X. Zhang H. Zhang W. Zhang P. Hao Y. Song R. (2016). Regulation of neuroendocrine cells and neuron-factors in ovary by zinc oxide nanoparticles. *Toxicol. Lett.* 256 19–32. 10.1016/j.toxlet.2016.05.007 27215404

[B23] Lv M. Huang W. Chen Z. Jiang H. Chen J. Tian Y. (2015). Metabolomics techniques for nanotoxicity investigations. *Bioanalysis* 7 1527–1544. 10.4155/bio.15.83 26168257

[B24] Mäkinen V. P. Soininen P. Forsblom C. Parkkonen M. Ingman P. Kaski K. (2008). 1H NMR metabonomics approach to the disease continuum of diabetic complications and premature death. *Mol. Syst. Biol.* 4 :167. 10.1038/msb4100205 18277383 PMC2267737

[B25] Parveen A. Rizvi S. H. Gupta A. Singh R. Ahmad I. Mahdi F. (2012). NMR-based metabonomics study of sub-acute hepatotoxicity induced by silica nanoparticles in rats after intranasal exposure. *Cell Mol. Biol. (Noisy-le-grand)* 58 196–203. 23273212

[B26] Pujalte I. Passagne I. Brouillaud B. Treguer M. Durand E. Ohayon-Courtes C. (2011). Cytotoxicity and oxidative stress induced by different metallic nanoparticles on human kidney cells. *Part Fibre Toxicol.* 8:10. 10.1186/1743-8977-8-10 21371295 PMC3058043

[B27] Ryan D. Robards K. (2006). Metabolomics: the greatest omics of them all? *Anal. Chem.* 78 7954–7958. 10.1021/ac0614341 17134127

[B28] Schaffer S. W. Azuma J. Mozaffari M. (2009). Role of antioxidant activity of taurine in diabetes. *Can. J. Physiol. Pharmacol.* 87 91–99. 10.1139/Y08-110 19234572

[B29] Schuller-Levis G. B. Park E. (2003). Taurine: new implications for an old amino acid. *FEMS Microbiol. Lett.* 226 195–202. 10.1016/S0378-1097(03)00611-6 14553911

[B30] Soininen P. Kangas A. J. Würtz P. Tukiainen T. Tynkkynen T. Laatikainen R. (2009). High-throughput serum NMR metabonomics for cost-effective holistic studies on systemic metabolism. *Analyst* 134 1781–1785. 10.1039/b910205a 19684899

[B31] Taylor N. S. Weber R. J. M. White T. A. Viant M. R. (2010). Discriminating between different acute chemical toxicities via changes in the daphnid metabolome. *Toxicol. Sci.* 118 307–317. 10.1093/toxsci/kfq247 20719749

[B32] Tuomela S. Autio R. Buerki-Thurnherr T. Arslan O. Kunzmann A. Andersson-Willman B. (2013). Gene expression profiling of immune-competent human cells exposed to engineered zinc oxide or titanium dioxide nanoparticles. *PLOS ONE* 8:e68415. 10.1371/journal.pone.0068415 23894303 PMC3718780

[B33] Wan Q. He Q. Deng X. Hao F. Tang H. Wang Y. (2016). Systemic metabolic responses of broiler chickens and piglets to acute T-2 toxin intravenous exposure. *J. Agric. Food Chem.* 64 714–723. 10.1021/acs.jafc.5b05076 26714875

[B34] Wang Z. L. (2008). Splendid one-dimensional nanostructures of zinc oxide: a new nanomaterial family for nanotechnology. *ACS Nano* 2 1987–1992. 10.1021/nn800631r 19206442

[B35] Xia T. Kovochich M. Liong M. Maedler L. Gilbert B. Shi H. (2008). Comparison of the mechanism of toxicity of zinc oxide and cerium oxide nanoparticles based on dissolution and oxidative stress properties. *ACS Nano* 2 2121–2134. 10.1021/nn800511k 19206459 PMC3959800

[B36] Yan G. Huang Y. Bu Q. Lv L. Deng P. Zhou J. (2012). Zinc oxide nanoparticles cause nephrotoxicity and kidney metabolism alterations in rats. *J. Environ. Sci. Health A Tox. Hazard. Subst. Environ. Eng.* 47 577–588. 10.1080/10934529.2012.650576 22375541

[B37] Yang X. Shao H. Liu W. Gu W. Shu X. Mo Y. (2015). Endoplasmic reticulum stress and oxidative stress are involved in ZnO nanoparticle-induced hepatotoxicity. *Toxicol. Lett.* 234 40–49. 10.1016/j.toxlet.2015.02.004 25680694 PMC4344938

[B38] Zhai G. Wang-Sattler R. Hart D. J. Arden N. K. Hakim A. J. Illig T. (2010). Serum branched-chain amino acid to histidine ratio: a novel metabolomic biomarker of knee osteoarthritis. *Ann. Rheum. Dis.* 69 1227–1231. 10.1136/ard.2009.120857 20388742

[B39] Zhao J. Xu L. Zhang T. Ren G. Yang Z. (2009). Influences of nanoparticle zinc oxide on acutely isolated rat hippocampal CA3 pyramidal neurons. *Neurotoxicology* 30 220–230. 10.1016/j.neuro.2008.12.005 19146874

[B40] Zhao Y. Feng Y. Li L. Zhang H. Zhang Y. Zhang P. (2016a). Tissue-specific regulation of the contents and correlations of mineral elements in hens by zinc oxide nanoparticles. *Biol. Trace Elem. Res.* 177 353–366. 10.1007/s12011-016-0847-4 27830451

[B41] Zhao Y. Li L. Zhang P. Liu X. Zhang W. Ding Z. (2016b). Regulation of egg quality and lipids metabolism by zinc oxide nanoparticles. *Poult. Sci.* 95 920–933. 10.3382/ps/pev436 26908885

