# Supplementary figures and images for: Zinc Oxide Nanoparticle Caused Plasma Metabolomic Perturbations Correlate with Hepatic Steatosis
Source: Front Pharmacol. 2018 Jan 30;9:57. doi: 10.3389/fphar.2018.00057 (PMC5810292; doi:10.3389/fphar.2018.00057)

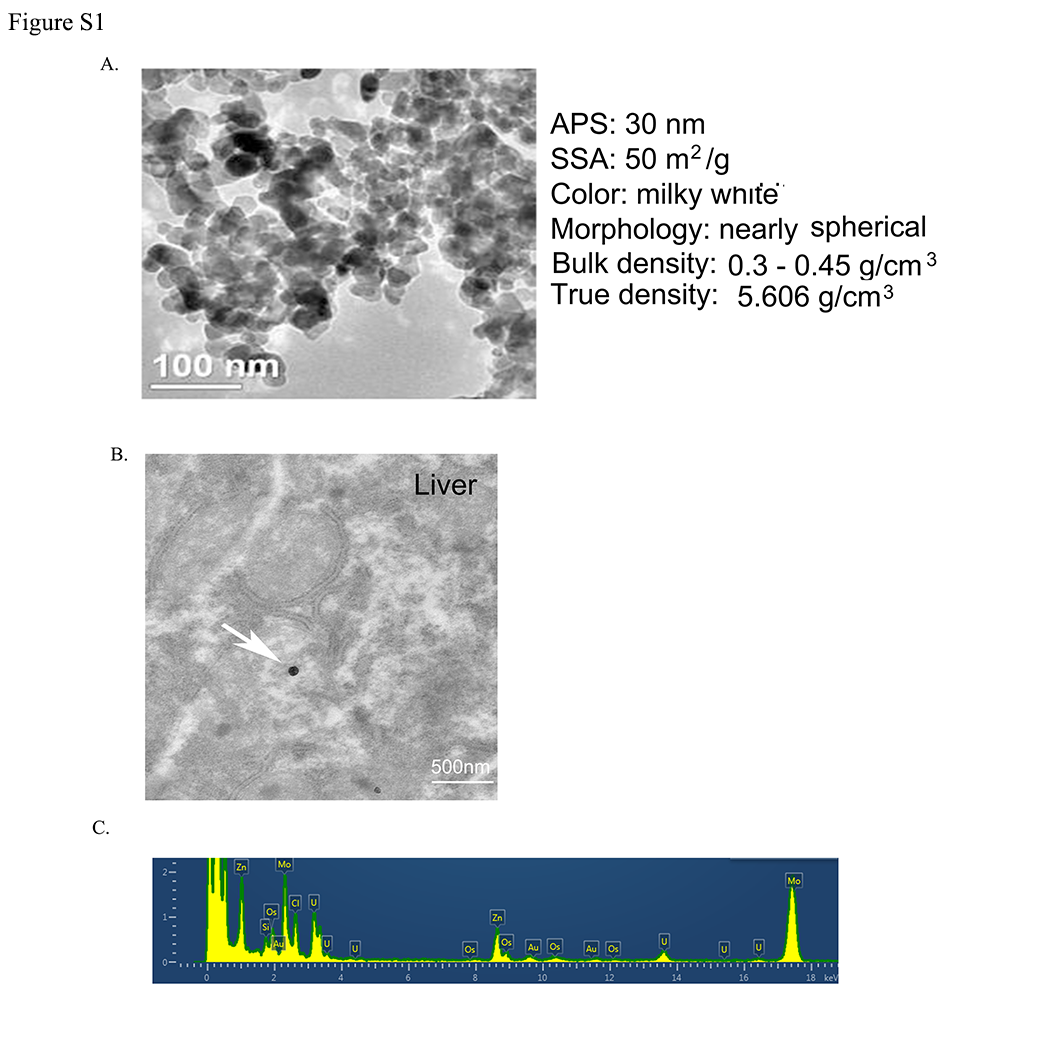

Supplement: Supplementary file 1 [file Image_1.TIF]

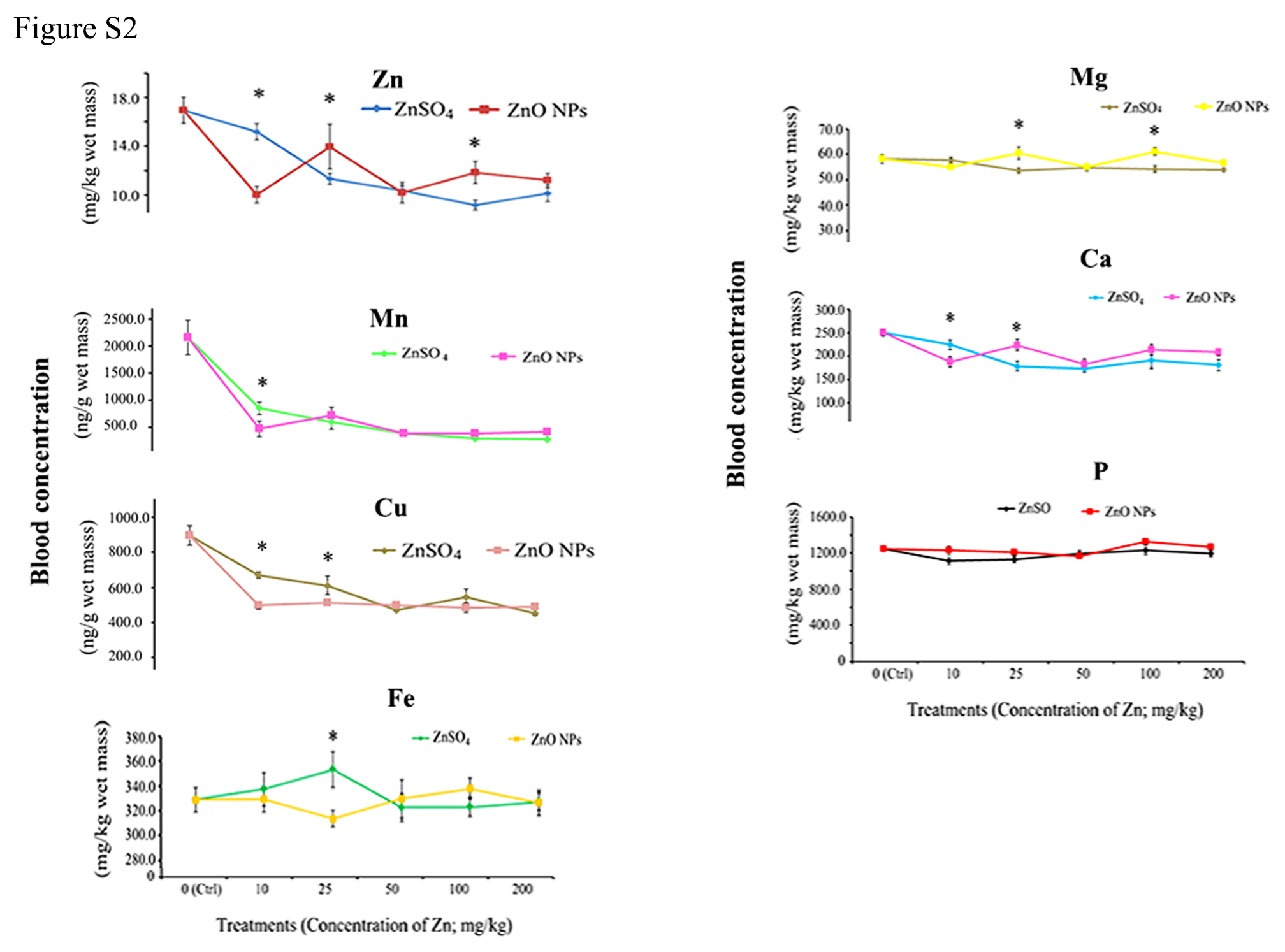

Supplement: Supplementary file 2 [file Image_2.TIF]
