# Supplementary material for: Zinc Oxide Nanoparticle Caused Plasma Metabolomic Perturbations Correlate with Hepatic Steatosis
Source: Front Pharmacol. 2018 Jan 30;9:57. doi: 10.3389/fphar.2018.00057 (PMC5810292; doi:10.3389/fphar.2018.00057)
Supplement: Supplementary file 3 [file Table_1.DOC]

Table S1 Ingredient composition of the basal diet for the layers.

| Item | Amount (6-18wks of age) | Amount (19-30wks of age) |
| --- | --- | --- |
| **Ingredient**  Corn, %  Soybean meal, %  Limestone, %  Vitamin-mineral premix^1^  Soybean oil  Total  **Nutrient level**  Metabolizable energy, (MJ/kg)  CP (%)  Calcium (%)  Nonphytate phosphorus (%)  Lysine (%)  Methionine (%)  Methionine + cysteine (%)  Zn (ppm) | 66.10  20.00  8.00  5.00  0.90  100.00  11.27  14.12 (14.25)^2^  3.21 (3.17) ^2^  0.33  0.69  0.31  0.62  11.98 | 62.10  24.00  8.00  5.00  0.90  100.00  11.58  15.98 (16.12) ^2^  3.11 (3.05) ^2^  0.31  0.72  0.36  0.66  12.03 |

^1^Provided per kilogram of product: 181,000 IU of vitamin A; 37,000IU of vitamin D_3_; 510 mg of vitamin E; 35 mg of vitamin K_3_; 33.5 mg vitamin B1; 72 mg vitamin B2; 16 mg vitamin B6; 0.5 mg vitamin B12; 280 mg d-pantothenic acid; 550 mg of niacin; 9 mg folic acid; 2.0mg d-biotin; 10,000mg choline; 1800 mg Mn; 2500 mg Fe; 500 mg Cu; 6.0 mg Se; 18,000mg Lysine; 20,000 mg Methionine; 20.0 mg I; 185.0g of Ca; 80 g of P; 100.0 g of NaCl. **No** **Zn was added in the premix**.

^2^The number in parentheses indicates the analyzed value.
