# Supplementary material for: Zinc Oxide Nanoparticle Caused Plasma Metabolomic Perturbations Correlate with Hepatic Steatosis
Source: Front Pharmacol. 2018 Jan 30;9:57. doi: 10.3389/fphar.2018.00057 (PMC5810292; doi:10.3389/fphar.2018.00057)
Supplement: Supplementary file 4 [file Table_2.DOCX]

Table S2 Primer sequences for q-RT-PCR

| **Gene** | **Site** | **Sequence** |
| --- | --- | --- |
| FASN | Forward | CTGTTCTCTCGTGTCCAAAGT (Sense) |
|  | Reverse | CGTGCAGCTCTGTTTCATTATC (Anti-sense) |
| Decr1 | Forward | CTGAAAGGAACGGCAGAAGAA (Sense) |
|  | Reverse | GGATGTCCTGCCACTTGAATTA (Anti-sense) |
| ELOVL1 | Forward | GTGTGGCTGTGGAGAATGTA (Sense) |
|  | Reverse | CCCACTCTGTACTAGGCATAAG (Anti-sense) |
| ELOVL5 | Forward | CTACTATGGATTGTCGGCTGTT (Sense) |
|  | Reverse | ACAGCTGGTCTGGAAGATTG (Anti-sense) |
| ELOVL6 | Forward | GCACGCCGTTATGTACTCTTA (Sense) |
|  | Reverse | TGATCTGCGACAAGGTGATG (Anti-sense) |
| ELOVL7 | Forward | GGTTACTCGTTCCGATGTGAT (Sense) |
|  | Reverse | GGAGAAGTAGTACAGCCAACAA (Anti-sense) |
| Gpam | Forward | GGCACACCTTGCACTATTAGA (Sense) |
|  | Reverse | TGAAGGACAGATGCCACAATAA (Anti-sense) |
| CYP51A1 | Forward | TCATCGCTGTATTGGAGAGAAC (Sense) |
|  | Reverse | GTAGCCATCGACGAGATCAAA (Anti-sense) |
| NSDH1 | Forward | GTGTGGGAAGGCATTTCATATC (Sense) |
|  | Reverse | GCCAGCCAGTAGGGAATATAG (Anti-sense) |
| DHCR7 | Forward | TTGGGCTTGGTTGGCTATTA (Sense) |
|  | Reverse | TCAATGTAGTCAGGCTTCTTCC (Anti-sense) |
