# Supplementary material for: Zinc Oxide Nanoparticle Caused Plasma Metabolomic Perturbations Correlate with Hepatic Steatosis
Source: Front Pharmacol. 2018 Jan 30;9:57. doi: 10.3389/fphar.2018.00057 (PMC5810292; doi:10.3389/fphar.2018.00057)
Supplement: Supplementary file 5 [file Table_3.DOC]

Table S3 Primary antibody information

| **Gene symbol** | **Name** | **Cat. #** | **Predicted size** | **Source (Animal)** | **Company** |
| --- | --- | --- | --- | --- | --- |
| Actin | Actin | ab3280 | 43 kDa | Mouse( monoclonal) | Abcam plc. |
| Caspas 8 | Caspas 8 | bs-0052R | 12/55 kDa | Rabbit (polyclonal) | Beijing Biosynthesis Biotechnology CO. |
| SREBF1 | Sterol Regulatory Element Binding Transcription Factor 1 | D121269 | 54/126 kDa | Rabbit(polyclonal) | Sangon Biotech(Shanghai) CO Ltd |
| FASN | Fatty Acid Synthase | D162701 | 272 kDa | Rabbit (polyclonal) | Sangon Biotech (Shanghai) CO.Ltd |
| LIPC | Human Hepatic Lipase Gene | Bs-18288R | 53 kDa | Rabbit(polyclonal) | Beijing Biosynthesis Biotechnology CO. |
| GLUD2 | Glutamate Dehydrogenase 2 | Bs-13390R | 111 kDa | Rabbit(polyclonal) | Beijing Biosynthesis Biotechnology CO. |
| ASAT | Aspartate Aminotransferase | Bs-3977R | 46 kDa | Rabbit(polyclonal) | Beijing Biosynthesis Biotechnology CO. |
| AMPD | AMP deaminase | Bs-2077R | 86 kDa | Rabbit(polyclonal) | Beijing Biosynthesis Biotechnology CO. |
| GPT2 | Glutamic pyruvic transaminase 2 | Bs-16302R | 58 kDa | Rabbit(polyclonal) | Beijing Biosynthesis Biotechnology CO. |
